# Should EU member states help each other? How the national context shapes individual preferences for European solidarity

**DOI:** 10.1057/s41295-022-00301-9

**Published:** 2022-04-28

**Authors:** Camilla Mariotto, Alessandro Pellegata

**Affiliations:** 1 Department of Political Science, University of Innsbruck, Universitätstrasse 15, 6020 Innsbruck, AT Austria; 2 Department of Social and Political Sciences, Università degli Studi di Milano, Via Conservatorio 7, 20122 Milan, IT Italy

**Keywords:** EU financial assistance, European solidarity, European Union, Eurozone crisis, International redistribution

## Abstract

With the outbreak of the Eurozone crisis, the idea of providing cross-national financial transfers to countries in economic and financial difficulties has exacerbated the political divide between EU member states with strong macroeconomic performances, which were only weakly hit by the crisis, and the countries of the Eurozone periphery that struggled with a harsh economic downturn. This paper aims to explain which factors drive public support for cross-national solidarity within and across countries. We argue that the national context in which citizens live affects their preferences for providing financial help to other European countries, and moderates the role played by subjective egotropic and sociotropic economic concerns, ideological predispositions, and Eurosceptic vote choices in shaping public support for European solidarity. Using the original REScEU 2016 survey, we find that subjective economic motivations provide a limited contribution in explaining support for European solidarity, and almost only in countries weakly hit by the crisis. On the contrary, left–right positions, and especially Eurosceptic vote choices, strongly polarize preferences for EU financial assistance, both within and across countries with voters from Eurosceptic parties more(less) likely to support European solidarity in countries strongly(weakly) hit by the Eurozone crisis.

**Supplementary Information:**

The online version contains supplementary material available at 10.1057/s41295-022-00301-9.

## Introduction

The Eurozone crisis forced the European Union (EU) to adopt new policies to help member states in economic and financial difficulties, and to safeguard the single currency’s stability. The harsh debates over the decision to bail out EU member states in crisis have exacerbated the conflicting views across countries on who needs to carry the burden of the adjustment costs. This new political divide especially separates the ‘frugal’ countries of Northern Europe that are fiscally conservative and which have strong macroeconomic performances from highly indebted Southern European countries, which were hit hardest by the crisis (Ferrera 2017; Matthijs and McNamara 2015). This conflict has been recently reinvigorated by the negotiations that surrounded the adoption of the Next Generation EU plan to counteract the economic and social consequences of the COVID-19 pandemic.

This paper investigates how public preferences for EU financial assistance vary across EU member states. The purpose is to assess how the national context in which citizens live moderates the heuristics on which they rely to shape support for European solidarity.

The recent literature has demonstrated that public attitudes towards European fiscal solidarity are mostly driven by economic self-interest, cosmopolitan attitudes, and political orientations. Individuals are in favour of cross-national redistribution if they perceive an economic benefit from it, if they identify themselves with the EU, and if they have positive attitudes towards immigration and transnational experiences (Bechtel et al. 2014; Ciornei and Recchi 2017; Daniele and Geys 2015; Kuhn and Stoeckel 2014; Verhaegen 2018). Furthermore, political orientations and partisan cues are crucial informational shortcuts when forming opinions on a complex policy issue like EU financial assistance (Bechtel et al. 2014; Daniele and Geys 2015; Kleider and Stoeckel 2019; Kuhn et al. 2018; Stoeckel and Kuhn 2018). Focussing on the role played by the national context, the literature provides mixed and inconclusive results. According to Lengfeld et al. (2015) and Vasilopoulou and Talving (2020), support for EU financial assistance is lower among citizens living in countries with poor macroeconomic performances, while Kuhn and Stoeckel (2014) and Daniele and Geys (2015) obtain opposite results. Kuhn and Stoeckel (2014) and Vasilopoulou and Talving (2020) have also explored how the national context moderates the association between individual level factors and public support for EU financial assistance, showing that poor macroeconomic performance dampens the impact of both socio-economic status and national/European identity.

Existing studies, however, fail to consider different forms through which intra-EU financial assistance can be provided, and the related salient debate on who should carry the burden of the adjustment costs. We expect that citizens who live in countries strongly hit by the Eurozone crisis tend to support genuine forms of European solidarity that are not contingent on austerity measures. On the contrary, those who live in countries weakly hit by the crisis tend to prefer the conditionality regime, which implies that recipient countries implement austerity measures and structural reforms in exchange for financial help, or even express opposition to any kind of financial assistance. Furthermore, we postulate that the national context moderates how subjective egotropic and sociotropic economic evaluations, ideological predispositions, and votes for Eurosceptic parties contribute to shape public support for European solidarity across countries. Egotropic economic evaluations and ideological predispositions should shape support for European solidarity only in countries weakly hit by the crisis, while sociotropic economic evaluations and Eurosceptic vote choices operate differently across EU member states. Individuals concerned about the national economy and those who voted for Eurosceptic parties should be more (less) likely to support European solidarity in countries strongly (weakly) hit by the crisis.

The present study seeks to expand previous findings by providing important contributions to our understanding of public support for European solidarity. First, by relying on novel data taken from the original REScEU 2016 survey conducted in six EU member states—France, Germany, Italy, Poland, Spain, and Sweden—in autumn 2016, we propose a new measurement of European solidarity that, in contrast with existing items, allows us to disentangle genuine preferences for European solidarity based on intra-EU redistribution mechanisms and support for bailouts under the conditionality regime implemented by the EU economic governance during the Eurozone (Ferrera and Pellegata 2016). This survey item also addresses the hotly debated issues on which actors were held more responsible for the crisis, and whether, and how, EU member states should share the burden of the adjustment costs.

Second, empirical results confirm only some of our expectations, revealing that citizens’ attitudes towards EU financial assistance are more complex than is often assumed in the debate, and that the explanatory power of the traditional heuristics on which citizens rely varies across EU member states. National party systems, and especially the positions taken by challenger parties, play a relevant role in moderating public support for European solidarity in all sample countries. We believe that the present study has relevant implications not only for the understanding of public attitudes towards EU integration, but also for the debate around the adoption of a fiscal union in the Eurozone.

## The asymmetric impact of the crisis and the divide on European solidarity

Different positions on the causes and consequences of the asymmetry between core countries of Northern Europe, with strong macroeconomic and financial performances, and Southern member states, struggling with excessive deficit and increasing public debt, were already at stake at the outset of the European Monetary Union (EMU) (Copelovitch et al. 2016; Pérez 2019).

Figure A1 in the online appendix shows the consequences of the crisis on main macroeconomic indicators across our sample countries. Italy and Spain experienced an alarming decline in GDP that made their economy enter recession. This downturn was exacerbated by a dramatic rise of the unemployment rate and a deterioration of the banking sector in Spain, and increasingly high government debt in Italy. Conversely, being a surplus country in the Eurozone, Germany faced, by comparison, substantially weaker and less direct problems, such as lack of external demand, financial market turbulence, and value losses in foreign assets. In Sweden, the impact of the crisis was relatively weak, and the recovery of the economy to pre-crisis levels was faster, thanks to its current account surplus and flexible exchange rate. In Poland, the current account deficits and the fast accumulation of foreign debt accompanied the boom in the year following EU accession. Being outside the Eurozone allowed the Polish government to devalue its currency in response to the balance-of-payment crisis (Walter et al. 2020). Finally, France was somehow between these two groups of countries because its macroeconomic performance before 2008 was not as good as in Germany, Poland, and Sweden, but it did not deteriorate after the crisis as was the case in Italy and Spain.

These macroeconomic divergence polarize political actors on whether (and how) the burden of the Eurozone crisis should be distributed among European member states and citizens, or should fall on the shoulders of single countries instead (Kriesi and Grande 2016). The concept of European solidarity refers to the individual willingness of states to share obligations and resources across EU countries and citizens to prevent, or redress, situations of economic, social, political, and environmental adversity (Ciornei and Recchi 2017: 470; Ciornei and Ross 2021: 210). As Zürn (2000: 199) states, the acceptance of redistributive policies is the best indicator for this. Thus, in EU economic governance, European solidarity can be measured with public support for providing EU financial assistance towards fellow member states that are facing severe economic or financial conditions. However, according to the deservingness theory (van Oorschot 2000), support for welfare programmes largely depends on individuals’ perceptions about whether—and to what extent—the target groups deserve to receive assistance. People tend to consider less deserving of help an individual or, in this context, a member state in economic difficulties when they perceive that they are responsible for their own situation (Vandenbroucke 2017: 21; Verhaegen 2018: 883).

By considering who was to blame for the crisis, two competing narratives about the willingness to adopt international redistribution mechanisms were in play across the EU. Most of the media and parties in Northern countries often framed the debate on the Eurozone crisis as a conflict between ‘northern saints’ versus ‘southern sinners’ (Matthijs and McNamara 2015). Hard work, prudent savings, moderate consumption, wage restraint, and fiscal stability were seen as northern virtues and were juxtaposed to the southern vices of low competitiveness, meagre savings, undeserved consumption, inflated wages, and fiscal profligacy which characterized the debtor countries (Matthijs and McNamara 2015). As these vices are seen as the main culprit for the declining competitiveness in the economies of peripheral countries, the burdens of fiscal adjustment should fall exclusively on their national taxpayers (Ferrera 2017). Therefore, Northern European countries, and more generally those that were weakly hit by the Eurozone crisis, preferred internal adjustment, and supported a strong conditionality regime, according to which adjustment costs on the debt-ridden countries should be accompanied by precise conditions for repayment, domestic structural reforms, and strong commitments to fiscal discipline.

In contrast, public opinion and a large part of the elites in Southern countries, which were strongly hit by the crisis and thus were more plausible recipients of financial assistance, blamed excessive rigour and lack of solidarity by the EU institutions and rich governments, and opposed austerity measures. Their governments called for more flexibility in the application of rules, the mobilization of EU resources for investment and growth, and, most importantly, debt mutualization in the form of Eurobonds or fiscal equalization schemes (Ferrera 2017; Schimmelfennig 2015). As Verhaegen (2018: 883) states, in countries hardly hit by the crisis, a sense of shared responsibility for the causes of it could result in a sense of a shared responsibility in dealing with the consequences as well, thus fostering public support for European solidarity (see also Conti et al. 2020).

### H1a

Individuals living in countries weakly hit by the crisis, such as Germany and Sweden, and to a lesser extent France and Poland, are less likely to support European solidarity by opposing any kind of EU financial assistance, or preferring EU financial programmes that are contingent on austerity measures*.*

### H1b

Individuals living in countries strongly hit by the crisis, such as Italy and Spain, are more likely to support European solidarity by means EU financial assistance programmes that are not contingent on austerity measures.

## The conditional role of the national context

The literature has proposed several individual-level explanations of public support for EU financial assistance in times of crisis (Bechtel et al. 2014; Gerhards et al. 2019). However, the role played by individual dispositions in shaping preferences for EU financial assistance may depend on common experiences with the economic and social consequences of the Eurozone crisis, and the positions taken by national parties and governments over the responsibilities of the EU. Therefore, we argue that the national context in which citizens live moderates how traditional heuristics contribute to explaining public support for fiscal solidarity across EU member states. We focus on subjective egotropic and sociotropic economic evaluations as well as political factors such as ideological predispositions and party choices.^1^

### Subjective economic evaluations

A broad literature argues that individuals’ opinions on economic policy depend on their expectations about how the proposed policy would affect their future earnings (see Bechtel et al. 2014; Gabel 1998). According to the economic self-interest approach, those who perceive that a specific measure will be detrimental for their economic status are less likely to support its introduction. This approach indicates that citizens in worse economic conditions, who were more exposed to personal losses during the crisis, should be less likely to support financial transfers to other EU member states in difficulties because they are concerned that national governments may decide to finance bailouts by raising taxes and/or reducing spending on domestic welfare transfers of which they are recipients.

However, we expect that this argument is valid only in those countries that were weakly hit by the economic downturn in which the crisis disproportionately impacted individuals with a low socio-economic status. The narrative portrayed by most of the media and political elites in these countries claimed that EU financial assistance provides benefits to individuals in profligate countries, at the expense of the population in virtuous ones. Therefore, individuals who have experienced economic losses after the onset of the crisis should be less likely to support forms of European solidarity that imply cross-national redistribution.

By contrast, in Southern European countries, like Italy and Spain, the economic and social impact of the crisis was much more severe spreading across diverse socio-economic groups, with the consequence that a large share of the population perceived their income as diminished compared to the past. Therefore, we expect that this diffuse feeling of economic losses induced a shared support for intra-EU financial assistance programmes that redistribute resources from richer to less affluent countries. The narrative portrayed in the countries hardly hit by the crisis reinforced this logic, by shifting the blame for the crisis to the EU institutions and demanding, therefore, that the same institutions should also deal with the consequences of the crisis (Verhaegen 2018).

#### H2a

In Germany and Sweden, and to a lesser extent France and Poland, individuals who have experienced economic losses during the crisis are less likely (than those who have not experienced economic losses) to support European solidarity, by opposing any kind of EU financial assistance, or preferring EU financial programmes that are contingent on austerity measures.

#### H2b

In Italy and Spain, individual self-interest is not associated with public preferences for EU financial assistance.

An abundant literature shows that besides individual self-interest, sociotropic concerns about the national economy are also important in shaping public attitudes towards European integration and solidarity (Hooghe and Marks 2005; Vasilopoulou and Talving 2020).

Considering the asymmetric impact of the Eurozone crisis across EU member states and the harsh debate on the role of the EU to counteract the crisis, we expect that the retrospective evaluations of the national economy shape public preferences for EU financial assistance differently across countries. Given the dramatic economic and social consequences that the crisis displayed in Italy and Spain, we expect that individuals who perceive that their country’s macro-economic performance has worsened during the crisis are more likely to support European solidarity, through grants or soft loans not contingent on fiscal austerity, than those who believe that national economic conditions improved or stayed about the same. They are more willing to believe that the EU should be responsible to counterbalance inequalities, which were not solved by the EMU, and which were strongly exacerbated by the Eurozone crisis.

An opposite relationship should occur in countries weakly hit by the crisis. Public support for European solidarity is more likely to decline when citizens perceive that their country’s national economy worsened during the crisis, because providing financial help to other EU member states would mean a reduction in the financial resources available for domestic assistance programmes (Bechtel et al. 2014). A negative evaluation of the national economic performance would strengthen the view that Southern countries deserve less financial assistance.

#### H3a

In Germany and Sweden, and to a lesser extent France and Poland, individuals are less likely to support European solidarity, by opposing any kind of EU financial assistance, or by preferring EU financial programmes contingent on austerity measures, as their concern about their country’s economic performance rises.

#### H3b

In Italy and Spain, individuals are more likely to support European solidarity by means of EU financial assistance programmes that are not contingent on austerity measures, as their concern about their country’s economic performance rises.

### Political orientations and partisanship

The literature has broadly argued about how citizens often rely on informational shortcuts to form their opinion on complex policy issues. Political orientations and partisanship are likely to drive citizens’ preferences for cross-national fiscal solidarity, as in the case of their attitudes towards European integration (Bechtel et al. 2014; Hooghe and Marks 2005). It is plausible that public opinion over EU financial assistance, which is a form of international redistribution, reflects the traditional left–right divide as the literature observes with respect to domestic redistributive policies. Left-wing parties and voters are more likely to commit themselves to international equality and solidarity, and be supportive of unconditional financial help towards troubled countries, while right-wing parties and voters are less likely to support financial redistribution by opposing any form of financial assistance, unless conditional on austerity measures.

However, we expect to find empirical support for this argument only in countries weakly hit by the crisis in which the issue of European solidarity has been strongly polarized along the traditional left–right divide. Conversely, we expect the voters and parties in Italy and Spain to be more likely to support intra-EU redistribution mechanisms, regardless of their ideological positions. In line with this view, Maatsch (2014) shows that in bailout countries during the Eurozone crisis all parties supported Keynesian measures to cope with the consequences of the crisis, despite their economic stances.

#### H4a

In Germany and Sweden, and to a lesser extent France and Poland, individuals are less likely to support European solidarity, by opposing any kind of EU financial assistance, or by preferring EU financial programmes contingent on austerity measures, as their political orientation moves to the right.

#### H4b

In Italy and Spain, left–right orientations are not associated with public preferences for EU financial assistance.

An alternative perspective argues that European solidarity may juxtapose voters of Eurosceptic and mainstream parties. The multiple crises that hit Europe after 2008 fuelled support for Eurosceptic parties, which have politicized the European integration process, and especially any forms of redistribution of the adjustment burden of the economic crisis, as well as redistributive mechanisms of migrants and asylum seekers across EU member states (Hobolt and De Vries 2016; Hobolt and Tilley 2016; Hooghe and Marks 2018; Hutter et al. 2018; Kriesi et al. 2012).

However, to fully understand how the juxtaposition between mainstream and Eurosceptic parties may shape individual preferences for European solidarity, we should also consider the asymmetric impact of the Eurozone crisis across Europe and different types of Euroscepticism (Braun et al. 2019; Hutter et al. 2018; van Elsas and van der Brug 2015). In Northern and Eastern European countries, which were weakly hit by the crisis, Euroscepticism mostly has cultural and political roots and is driven by anti-immigration sentiments and the opposition to the increasing policy-making role of EU institutions that limits national sovereignty. Therefore, Eurosceptic voters and parties oppose EU financial assistance because it implies a redistribution of resources to other countries at the expense of fellow nationals. In Southern Europe, EU-related issues instead tend to merge with the economic dimension (Otjes and Katsanidou 2017). Eurosceptic voters and parties instead of opposing the integration process as such were motivated by a revision of the present framework of the EU and the prevailing economic policies. Shifting the blame for the crisis to EU institutions and core countries, Eurosceptic parties in the South thus called for more European solidarity through a mutualization of public debt among EU member states (Hobolt and Tilley 2016; Hutter et al. 2018). While this position was clearly taken by the radical-left party *Unidas-Podemos* (UP) that, at the time of the survey, was the unique (soft) Eurosceptic party in the Spanish party system, in Italy several parties with different ideological positions coexisted. *Movimento 5 Stelle* (M5S) is a populist party, whose leaders and supporters, while refusing to locate on the left–right spectrum, expressed ambiguous positions regarding the EU (Mosca and Tronconi 2019). During the Eurozone crisis M5S campaigned for a referendum on the Euro but, at the same time, claimed a ‘*return to the principles of solidarity and community*’ in the EU (Della Porta et al. 2017: 132). *Lega Nord* (LN) and *Fratelli d’Italia* (FdI) are instead radical-right parties that strongly opposed the EU fiscal austerity, but with “equivocal” positions towards the EU and European integration (Heinisch et al. 2021). Given the ambivalent positions of the Italian parties, we expect to find less straightforward results in Italy compared to Spain.

#### H5a

In Germany and Sweden, and to a lesser extent France and Poland, voters of Eurosceptic parties are less likely to support European solidarity (than those of mainstream parties), by opposing any kind of EU financial assistance.

#### H5b

In Spain, and to a lesser extent Italy, voters of Eurosceptic parties are more likely to support European solidarity (than those of mainstream parties), by means EU financial assistance programmes that are not contingent on austerity measures,.

## Data and methodology

We perform our analysis by employing data taken from the REScEU 2016 survey (Ferrera and Pellegata 2016). This survey was conducted in autumn 2016 in national samples of six EU member states—France, Germany, Italy, Poland, Spain, and Sweden—which, as already described above, were asymmetrically affected by the Eurozone crisis. The online appendix provides more information on the survey structure and the sampling design employed.

Our dependent variable—*EU financial support—*measures individuals’ support for cross-national financial transfers through a survey item that asks respondents to choose one of the six available options on whether and how the EU should provide financial help to member states in economic and financial difficulties. The wording is as follows:

During the recent Eurocrisis, a number of Member States in severe economic and financial conditions have asked for help from the EU. This has led to the adoption of new common rules on the provision of financial support to heavily indebted countries.

Please, indicate which of these statements comes closest to your view. Financial support from the EU should…

1. …not be a task for the EU to deal with;
2. …not be provided because Member States should take responsibility for their own problems instead of asking money from foreign taxpayers;
3. …be offered voluntarily only by those countries that consider it to be in their national interest;
4. …be accompanied by precise conditions for repayment and domestic policy reform, so as not to put the Monetary Union at risk;
5. ….take the form of soft loans, because all Europeans are in the same boat;
6. …be granted without conditions, in the name of solidarity between EU citizens and states.

While we are aware that this survey item is not free from caveats, and can be cognitively stressful, especially for respondents with low educational levels and those minimally interested in politics, or not at all, our indicator has two main strengths compared to the survey items used in previous studies. First, our question does not investigate public preferences for specific policy measures like bailouts and Eurobonds. Since these policies can be seen as (potentially) profitable investments, it is presumed that the rationale that lies behind their support is linked to the economic interest, rather than a readiness to help countries in difficulty (Bechtel et al. 2014; Daniele and Geys 2015). Second, in contrast to the item previously included in the 2010 and 2011 Eurobarometer and the 2014 European Election Survey, our question does not simply tap respondents’ general support for financial help, but also in which conditions financial help should be given. This choice allows us to disentangle a genuine sentiment of solidarity (as indicated in response categories 5 and 6) from support for the conditionality regime (as in category 4), which implies the imposition of fiscal austerity for recipient countries, and from a limited to a firm opposition to an institutionalized mechanism for EU financial assistance (as in categories 1, 2 and 3).

**Fig. 1:**
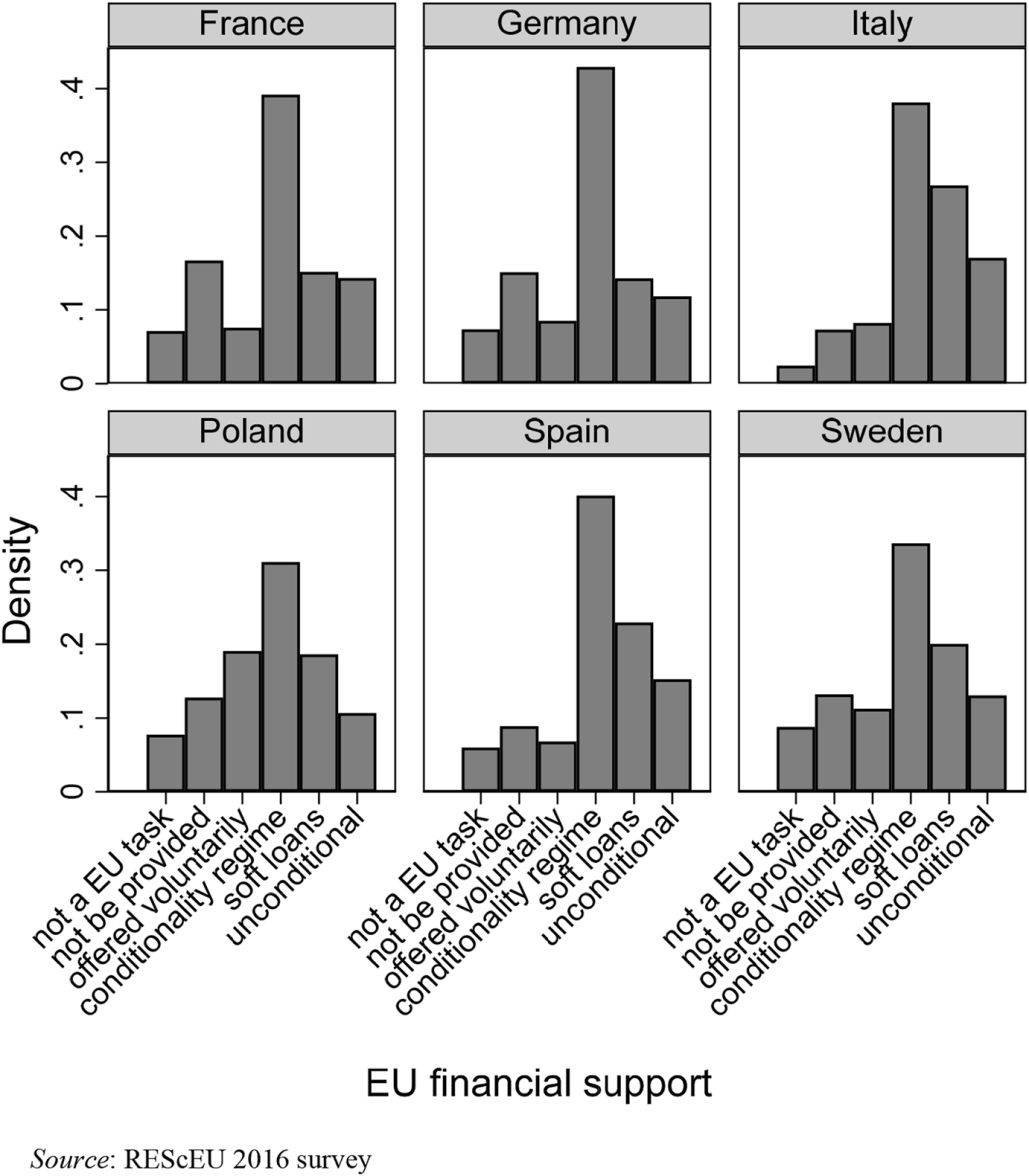
Distributions of preferences for EU financial support by country

The six graphs in Fig. 1 plot the distribution of public preferences for EU financial assistance in each sample country. Option 4, which represents the conditionality regime supported by the EU economic governance during the Eurozone crisis, is preferred by the majority of respondents in all sample countries. Not surprisingly, support for the conditionality regime is particularly high in Germany (45 per cent), which was the leading country during negotiations over the recent reform of the EU fiscal governance. In France and Germany, a severe (1) to a limited (3) opposition towards solidarity is preferred by a higher share of respondents than in peripheral countries. Non-Eurozone countries—Poland and Sweden—follow a similar pattern. Support for the conditionality regime is lower, and preferences for EU financial assistance are more polarized than in the Eurozone countries. However, in both Poland and Sweden the share of respondents who oppose European solidarity is very similar to that which has been detected in France and Germany, and is higher than in Italy and Spain. Despite the fact that the Italian and Spanish respondents are mostly supportive of financial transfers if bailouts were linked to economic conditionality, the distribution is skewed towards options (5) and (6). Indeed, more than 43 per cent in Italy and 38 per cent in Spain have chosen those options indicating the loosest forms of financing.

Now we turn to briefly illustrate the main explanatory factors referring to the online appendix for a more detailed description of coding rules adopted. Economic self-interest is operationalized with subjective *egotropic concerns*, a dummy variable coded 1 for respondents who think that their household financial situation worsened in the five years before the survey, and 0 for those who believe it stayed about the same or improved. Similarly, *sociotropic concerns* are measured through a dummy variable coded 1 for respondents who perceive the national economic conditions as having worsened compared to the previous five years and 0 otherwise. We estimate ideological leanings with the traditional individuals’ self-placement on the 0–10 *left–right* scale, where 0 means left and 10 means right. In the regression models, we employ its standardized transformation. Finally, vote choice for *Eurosceptic* parties is measured using a dummy variable coded 1 if a respondent in the last national election voted for a Eurosceptic party and 0 otherwise.^2^

We include several control variables that are plausible correlates with attitudes towards European solidarity. First, considering the relevance of cosmopolitan attitudes and transnational traits in explaining public support for different forms of European solidarity (Bechtel et al. 2014; Ciornei and Recchi 2017; Kuhn et al. 2018), we control for respondents’ *trans-EU experiences* through a dichotomous variable, coded 1 if individuals have ever visited another EU country for work, study, or family affairs and 0 otherwise. Second, we control for *occupational class* and *income*, which contribute to define respondents’ socio-economic status. Finally, *age*, *gender*, and *education* provide a better characterization of the demographic profile of respondents.

Given the non-ordinal nature of the dependent variable, the empirical analyses that follow employ multinomial regression models in which public attitudes towards EU financial support are regressed on the main independent variables and controls.^3^ First, we have run a model including only country dummies and socio-demographic variables—age, gender, education, occupation, and income—to detect variation in average public preferences for EU financial support across countries. Then, in the other four regression models, we have interacted country dummies with egotropic concerns, sociotropic concerns, ideological predispositions, and Eurosceptic vote choices, respectively, to account for the conditional role of the national context in moderating the associations between individual-level factors and public preferences for EU financial support.

## Empirical results

Our findings confirm that public preferences for EU financial support vary across sample countries, and that the national context moderates the role played by the traditional heuristics in shaping individual support for European solidarity. Overall, findings confirm only some of the hypotheses advanced, and reveal a scenario that is more complex than what we have postulated.

To allow a meaningful interpretation of the empirical results, we present and discuss a series of figures displaying average marginal effects that indicate the change in the predicted probability that respondents choose one of the response categories of the dependent variable at different values of the covariates. Positive values indicate that an increasing value, or a discrete change of the independent variable, is associated with a higher likelihood that respondents choose a specific form of EU financial support, while negative values indicate a lower likelihood. Ninety-five per cent confidence intervals are displayed. The online appendix reports the multivariate models from which marginal effects are computed.

**Fig. 2:**
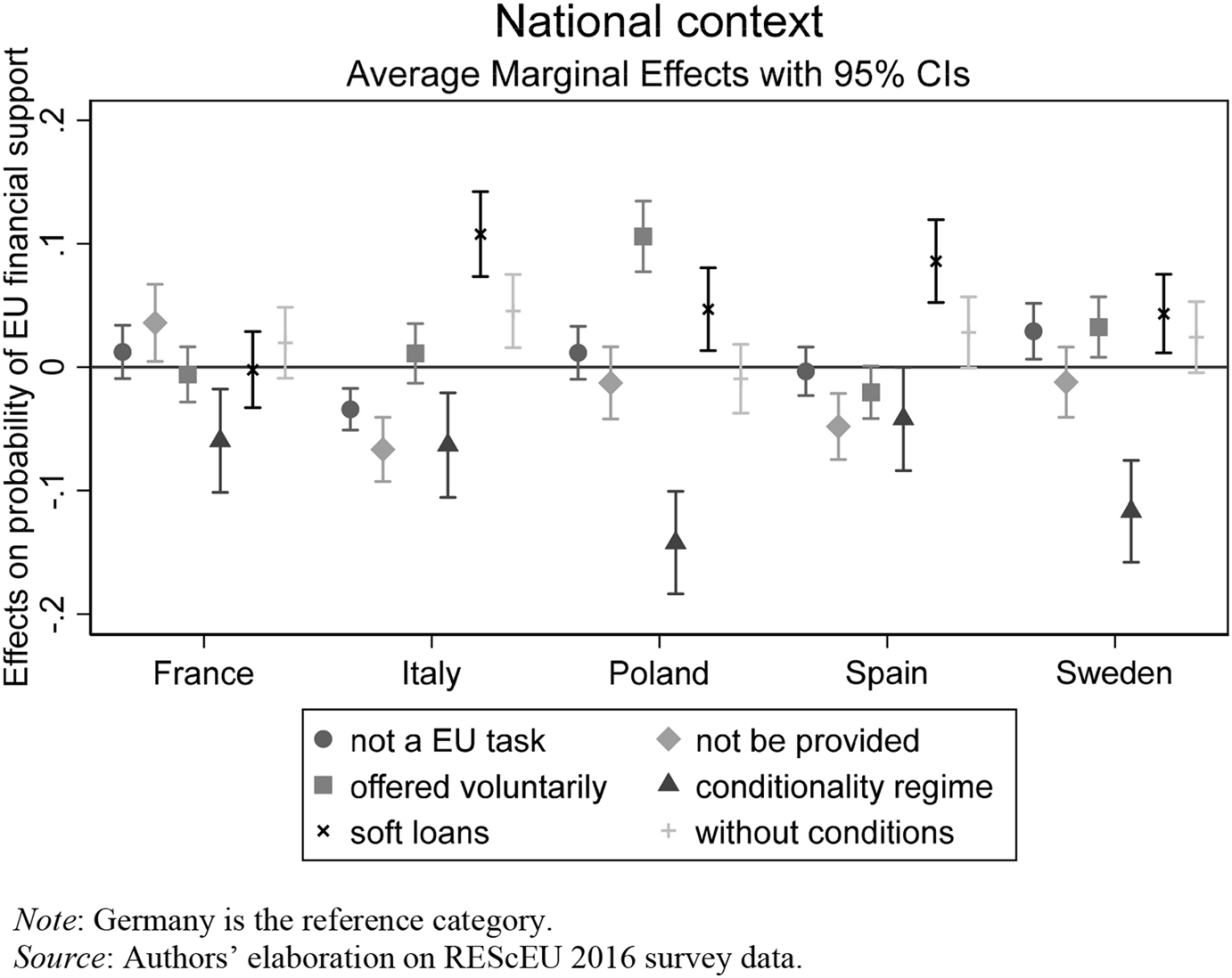
Association between countries and EU financial support

Figure 2 plots how the respondents’ probability of choosing one of the response categories of *EU financial support* changes across countries when compared to Germany, which is taken as the reference category because of its leading role in the negotiations over the bailout agreements during the Eurozone crisis.

Compared to German respondents, whose government was the main sponsor of the conditionality regime, respondents from all the other sample countries have a lower probability of preferring this option. As expected by H1a, the French public opinion is only slightly more likely than the German one to oppose European solidarity in believing that financial support should not be provided to countries in crisis. Polish and Swedish respondents instead are more polarized than Germans over the issue of EU financial assistance. This is not surprising given that they are neither members of the Eurozone or of the European Stability Mechanism (ESM). While they are more likely to prefer that each country could voluntarily decide to offer financial help to other member states in economic difficulties and, in the case of Sweden, to believe that bailing out EU member states in crisis should not be an EU task, they are also more likely to support cross-national solidarity mechanisms based on soft loans. Overall, empirical results provide evidence in support of H1b, but interesting variations also occur between Italy and Spain. Both Italians and Spaniards are less likely than Germans to believe that financial help to troubled countries should not be provided, and more likely to support European solidarity via soft loans. However, the preference for unconditional grants displays strong significance among Italian respondents, but weak corroboration (*p* < 0.1) among Spanish respondents.

**Fig. 3:**
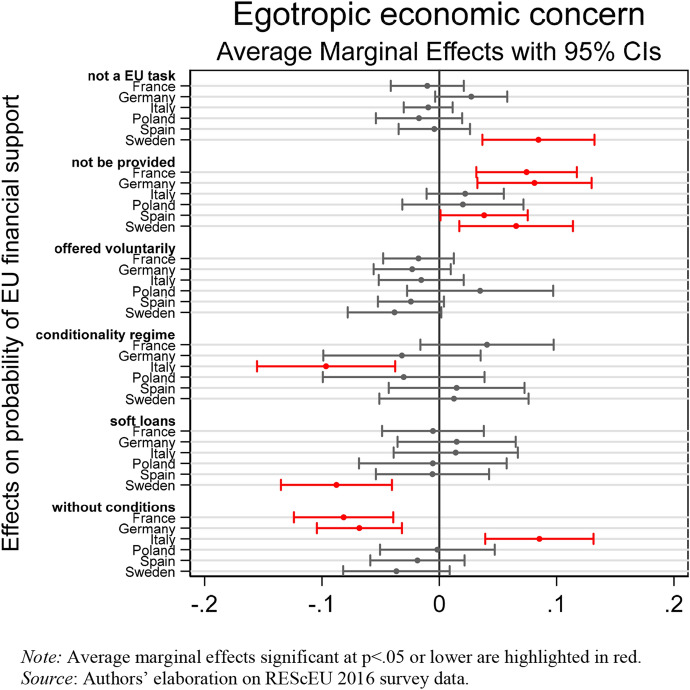
Association between egotropic economic concerns and EU financial support. (Color figure online)

We now move on to discussing the conditional effect of the national context on the association between subjective economic evaluations and preferences for EU financial assistance. Starting with the egotropic economic concerns, Fig. 3 shows the marginal effects of the interaction between respondents’ belief that their household financial situation had worsened after the outbreak of the crisis and country dummies on preferences for EU financial assistance.

In line with H2a, French, German, and Swedish citizens feeling more economically insecure are more likely to believe that financial assistance should not be provided, or even be an EU task (though only in Sweden). At the same time, they tend to oppose European solidarity mechanisms based on soft-loans (Sweden) or on unconditional grants (France and Germany). In Poland, instead, egotropic economic concerns are not associated with preferences for intra-EU financial help. In Southern Europe, we observe mixed results that are contrary to H2b. In Spain, respondents’ egotropic concerns are significantly—though weakly—associated with a higher propensity to oppose European solidarity, while in Italy they are associated with a higher propensity to support unconditional financial help. Probably, the concrete experience of a partial bank bailout made Spanish respondents with higher economic difficulties more sceptical of further foreign financing than Italian ones, regardless of its implementation (Walter et al. 2020).

Focussing on the sociotropic dimension, empirical results provide evidence that concerns about the national economy are (negatively) associated with preferences for EU financial assistance only in countries weakly hit by the crisis, especially in Northern Europe.

**Fig. 4:**
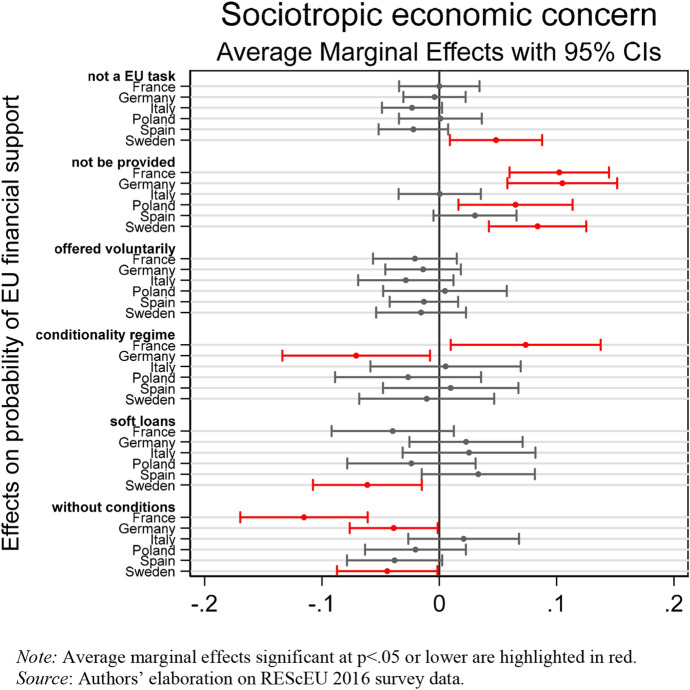
Association between sociotropic economic concerns and EU financial support. (Color figure online)

Figure 4 shows the marginal effects of the interaction between respondents’ belief that their country’s macroeconomic performance was worsened after the Eurozone crisis and country dummies on public preferences for EU financial assistance. In line with H3a, in France, Germany, Poland, and Sweden citizens who are more concerned about the situation of the national economy tend to oppose European solidarity by agreeing that the EU should not provide financial help to countries in economic and financial difficulties or even that this should be an EU task (Sweden). At the same time in France, Germany, and Sweden, but not in Poland, being concerned about the national economic situation is less likely to be associated with support for European solidarity through unconditional grants or soft loans (in Sweden only). In addition, despite German respondents on average tending to prefer the conditionality regime (see Fig. 1), when their concern about their own country’s economic performance rises, the probability of preferring such policy options decreases. This result confirms the domestic popular opposition Merkel’s government faced because of citizens’ concern about national macroeconomic performance. In France, on the contrary, respondents concerned about their country’s economy are more likely to sponsor the conditionality regime.

We do not find corroborating evidence for H3b. Italian and Spanish respondents who are concerned about their country’s economy are not significantly more likely to support European solidarity via soft loans or unconditional grants than their fellow nationals, who perceive their national economy as improved or stayed about the same in the five years before the survey was in field. This result can be plausibly explained by the fact that in these countries, there are a majority of citizens who are concerned about their national economic situation.

**Fig. 5:**
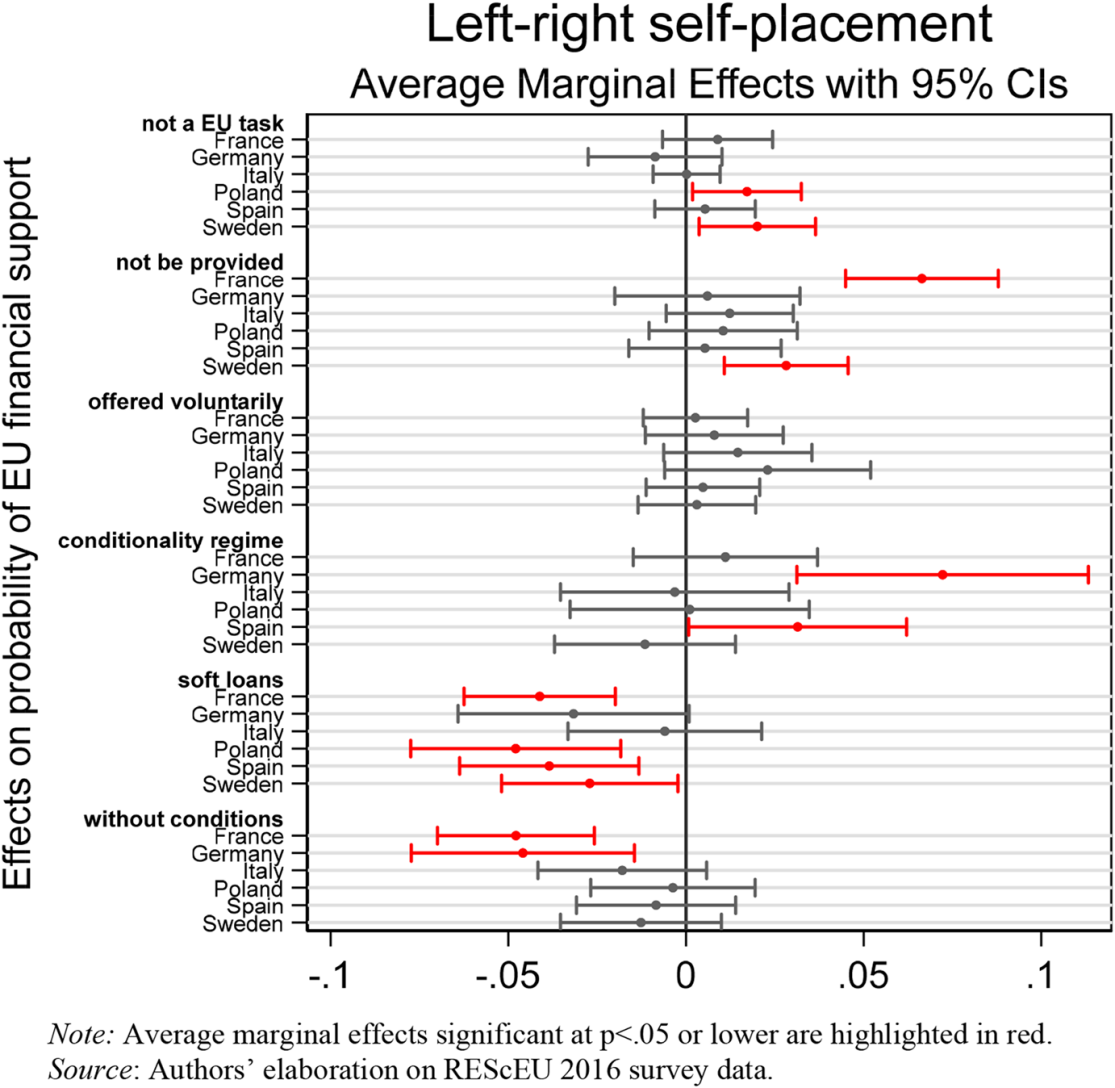
Association between ideological predispositions and EU financial support. (Color figure online)

We now turn to the empirical results regarding the association between political factors and preferences for EU financial assistance. Marginal effects of the interaction between left–right self-placement and country dummies are reported in Fig. 5 and indicate the change in the predicted probability of choosing each single category of the dependent variable for a one-standard deviation change in the left–right self-placement.

As expected in H4a, in France, Germany, Poland, and Sweden, right-wing voters are less likely to support European solidarity via soft loans and/or grants not conditional on austerity measures than centrist and left-wing voters. However, while French, Polish, and Swedish right-wing voters are also more likely to believe that financial assistance to EU member states in crisis should not be provided and/or be an EU task, German ones tend to support the conditionality regime. This result confirms that public support for this policy stems especially from right-wing voters who represent the electorate of the Merkel government. Instead, we obtained mixed results in Southern EU member states that confirm H4b in Italy, but not in Spain. As expected, in Italy the average public support for European solidarity is not affected by voters’ ideological leanings. On the contrary, the distribution of preferences in Spain resembles what we detect in Germany. Right-wing voters are more likely to prefer the conditionality regime and less likely to support financial assistance through soft loans. This result can be plausibly explained by the fact that during the Eurozone crisis, the issue of EU financial assistance was much more polarized in Spain than in Italy. In Spain, during the bailout negotiation, Rajoy’s right-wing government also confronted strong opposition from the radical-left anti-austerity movement of *Indignados* and the Spanish employer associations, who feared that such Troika-monitored bailouts would only exacerbate the crisis, and prolong the Spanish recession. In Italy, however, the pressures coming from the market, the EU institutions and the French and German governments forced Berlusconi’s right-wing cabinet to resign.

**Fig. 6:**
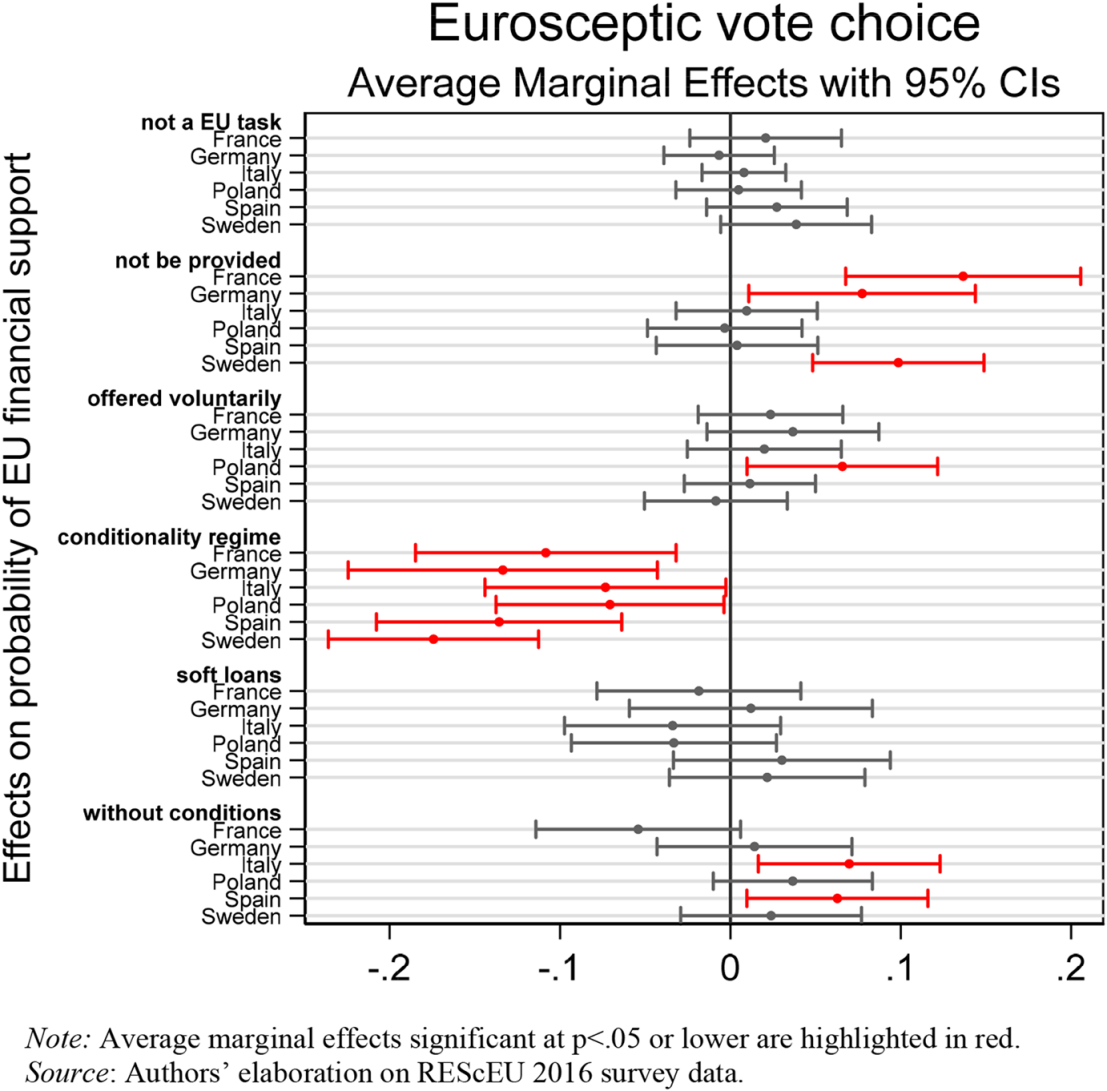
Association between Eurosceptic vote choices and EU financial support. (Color figure online)

Finally, Fig. 6 displays the marginal effects of the interaction of vote choices for Eurosceptic parties and the country dummies on public preferences for EU financial assistance. Empirical results obtained lend support to both H5a and H5b.

Given that Eurosceptic parties have contributed to polarizing the issue of solidarity between EU member states, it is not surprising to find that in all sample countries, voters of Eurosceptic parties are less likely to support the conditionality regime than voters of mainstream parties. However, as expected, a mediating role of the national context is at stake here. According to H5a, we have found that in France, Germany, and Sweden, respondents who voted for Eurosceptic parties—*Front National* (FN), *Alternative für Deutschland* (AfD) and *Die Linke*, and *Sverigedemokraterna* and *Vensterpartiet*, respectively—are more likely to believe that the EU should not provide any financial assistance to member states in crisis. Results obtained in Germany and Sweden confirm that, after the onset of the Eurozone crisis, the relationship between left–right positions and preferences for EU financial assistance resembles an inverted U-shaped curve. Voters and parties at the ideological extremes are significantly less likely than mainstream ones to support European solidarity (van Elsas and van der Brug 2015). In Poland, voters of the three right-wing Eurosceptic parties—*Prawo i Sprawiedliwos* (PiS), *Kukiz15*, and *KORWiN*—are more likely to prefer that EU member states voluntarily decide to offer financial support to other countries in crisis.

Conversely, in Italy and Spain voters of Eurosceptic parties in the last national election are more likely to support European financial aid without the prescription of conditionality than mainstream parties’ supporters. Interestingly, this result is straightforward both in Spain, where the only Eurosceptic party present at the time of the survey was the radical-left UP, and in Italy, where the populist M5S and the radical-right LN and FdI harshly criticized EU economic governance.

## Conclusions

The willingness to help other EU member states in crisis through international redistribution represents a highly contentious issue with regard to European public opinion. This paper has investigated how national contexts in a sample of six EU member states, with different macroeconomic performances and political positions on EU economic governance, contribute to shape public preferences for EU financial assistance and moderate the role played by traditional heuristics in explaining public support for European solidarity.

We believe that this study offers important contributions to the existing literature on public attitudes towards European solidarity, and more generally on EU support in correspondence of disruptive events, such as the Eurozone crisis, the refugee crisis, and the COVID-19 pandemic, that are threatening the very survival of the European project. First, our findings show that to fully understand public preferences for EU financial assistance within the EU, we need to disentangle genuine forms of solidarity between EU member states, which imply redistribution of resources from richer to less affluent countries, from the conditionality regime implemented through the ESM, which provides financial assistance in exchange for austerity measures and structural reforms for recipient countries.

Second, within-country variation in support of European solidarity is strongly intertwined with between-country variation. Empirical results obtained contradict Vasilopoulou and Talving (2020), who found that in poorer countries individual preferences in favour of financial assistance to EU member states in crisis are lower than in richer countries, by showing that public support for European solidarity tends to be higher in countries hardly hit by the crisis than in those with strong macroeconomic performances. Our findings, however, reveal a more nuanced scenario than those which have been commonly proposed by the extant literature. Compared to Germany, on average public opinion in all the other sample countries is less likely to favour the conditionality regime. Both French respondents, as well as Polish and Swedish ones, are more likely than Germans to oppose any kind of EU financial assistance. However, respondents in non-Eurozone members are polarized between opposing financial help and supporting European solidarity via soft loans to troubled countries. We have also detected interesting variations in countries strongly hit by the crisis. Public support for European solidarity is higher in Italy than it is in Spain, where the experience of a bailout (although it was limited to the banking system), and the related conditionality regime, might have produced as a response a general sentiment of distrust against any sort of EU financial assistance.

Third, the national context in which citizens live moderates the traditional heuristics they use to form their attitudes towards the EU and the specific issue of European solidarity. The explanatory power of both egotropic and sociotropic subjective economic motivations is weaker than what we have postulated, and is almost limited to north-western countries—France, Germany, and Sweden. Here individuals more worried about their household situation and/or the national economy are more likely to oppose any form of EU financial assistance. Only in Italy egotropic concerns are partially associated with a higher support for solidarity. On the contrary, public preferences for international redistribution are strongly associated with individuals’ left–right predispositions, with the exception of Italy, and even more with Eurosceptic vote choices. The asymmetric consequences of the Eurozone crisis across EU member states polarized voters and parties’ positions on EU financial assistance notwithstanding their stances on economic issues. Eurosceptic voters tend to support European solidarity in Italy and Spain, but to oppose any kind of financial assistance to troubled countries in France, Germany, Poland, and Sweden. This result confirms that the conflict over European solidarity has been mostly driven by challenger parties.

Finally, we believe that this study also provides relevant political implications on potential reforms of the economic governance of the Eurozone, especially after the outbreak of the COVID-19 pandemic, which has brought the issue of European solidarity back to collective attention. Baccaro et al.’s (2021) findings resonate with our empirical results by demonstrating that most Italian voters would choose to remain in the Euro if a bailout does not involve conditionality, but the pro-Euro majority turns into a plurality for ‘Italexit’, if the bailout is contingent on austerity policies.

We conclude by describing how further research can improve our results. Given that we have conducted our empirical analyses on a sample composed of only six countries, we need to be very careful in generalizing our results across Europe. Therefore, testing our hypotheses on larger samples would be extremely relevant to fully understand how public support for European solidarity changes across countries. Furthermore, future studies should take into consideration the important policy innovations implemented by Next Generation EU.

## Supplementary Information

Below is the link to the electronic supplementary material.

Supplementary file1 (DOCX 251 kb)

## Footnotes

1 Kuhn and Stoeckel (2014) and Vasilopoulou and Talving (2020) provide evidence that the national context also moderates the association between European identity and public support for European solidarity. Unfortunately, we cannot test this conditional expectation because our dataset does not include items measuring European identity or cosmopolitan attitudes.

2 We have relied on https://popu-list.org/ to define Eurosceptic parties. See the online appendix for the list of Eurosceptic parties across sample countries.

3 We have selected option 4 on the conditionality regime as a reference category in all multinomial regression models.
