# Supplementary material for: Should EU member states help each other? How the national context shapes individual preferences for European solidarity
Source: Comp Eur Polit. 2022 Apr 28;21(1):42–63. doi: 10.1057/s41295-022-00301-9 (PMC9047576; doi:10.1057/s41295-022-00301-9)
Supplement: Supplementary file 1 — Supplementary file1 (DOCX 251 kb) [file 41295_2022_301_MOESM1_ESM.docx]

Online Appendix for

*Should EU Member States Help Each Other?
How the National Context Shapes Individual Preferences for European Solidarity*

Table of contents

[1. The REScEU 2016 Survey 2](#_Toc91759381)

[2. The impact of the Eurozone crisis in sample countries 3](#_Toc91759382)

[3. Coding of independent variables and controls 4](#_Toc91759383)

[4. Descriptive statistics 6](#_Toc91759384)

[5. Regression models 8](#_Toc91759385)

[6. Robustness checks (results from models in single countries) 18](#_Toc91759386)

[7. References 22](#_Toc91759387)

## The REScEU 2016 survey

The REScEU 2016 survey was conducted in September-November 2016 on representative samples of adult citizens (18+ years old) in six countries: France (N=1,320), Germany (N=1,322), Italy (N=1,320), Poland (N=1,344), Spain (N=1,354) and Sweden (N=1,346). National samples of the REScEU 2016 Survey were built through a quota sampling around gender, age, educational level and NUTS1 macro-area of residence. A mixed method adopting CAWI and CATI methodology has been applied. The CAWI survey was administered to national stratified samples of at least 1,000 respondents, while the CATI survey was addressed to national samples of 320 respondents per country aged 55 or older to correct the inevitable underrepresentation of the older segments of the population with a lower access to internet. A separate section, which is not relevant for our analysis, of the REScEU 2016 survey also includes the United Kingdom but the attention was drawn on the Brexit referendum only.

## The impact of the Eurozone crisis in sample countries

#### Figure A1. Macroeconomic performance during the crisis across sample countries

*Source*: Authors’ elaboration on Eurostat data (https://ec.europa.eu/eurostat).

## Coding of independent variables and controls

*Gender*: (male = 0) (female = 1).

*Age*: (18-34 years old = 1) (35-54 years old = 2) (55 years old or more = 3).

*Education*: (up to lower secondary = 1) (higher secondary, advanced vocational training, lower tertiary education = 2) (higher tertiary education = 3).

*Occupational class:* this variable is a revised version of the Erikson and Goldthorpe (1992) class scheme that takes into account the works of Oesch (2006; 2008). It distinguishes between five different categories: (Employers and self-employed = 1) (salaried middle class = 2) (socio-cultural professionals = 3) (service and production workers = 4) (welfare recipients = 6). Our scheme makes three main revisions to the original one. First, we merged some original occupational classes into a single category (e.g. large employers and small-business owners; managers and technical; blue collars and service workers) to increase their relative size within the dataset and capture the differences we are mostly interested in. Second, we account for fissures within the post-industrial ‘service class’ by distinguishing socio-cultural (semi-)professionals (namely medical doctors, nurses and teachers) from the rest of the salaried middle class (Kitschelt and Rehm 2014). Finally, following Lepsius (2009), we introduce ‘welfare recipients’ (pensioners and unemployed workers receiving a subsidy) as a separate class. The other respondents for whom neither the Erikson and Goldthorpe (1992) nor the Oesch (2006) scheme were applicable (students, homemakers, unemployed) were grouped in two different categories: (unemployed = 5) (other inactive = 7).

*Income*

“What is your total gross household income before taxation?” (12 categories; 1=Lowest 12=Highest)

*Transnational experiences*

“Have you ever visited another EU country for work, study or leisure?”

1. Yes
2. No

Recoded: (1 = 1) (2 = 0)

*Egotropic economic concerns*

“Looking back over the last 5 years or so, would you say the financial situation of your household has improved a lot, improved somewhat, stayed about the same, got somewhat worse or got a lot worse?”

1. It has improved a lot
2. It has improved somewhat
3. It has stayed about the same
4. It has got somewhat worse
5. It has got a lot worse

Recoded: (1+2+3=0 “not concerned”) (4+5=1 “concerned)

*Sociotropic concerns*:

“Looking back over the last 5 years or so, would you say that the situation of the national economy in (OUR COUNTRY) has improved a lot, improved somewhat, stayed about the same, got somewhat worse or got a lot worse?”

1. It has improved a lot
2. It has improved somewhat
3. It has stayed about the same
4. It has got somewhat worse
5. It has got a lot worse

Recoded: (1+2+3=0 “not concerned”) (4+5=1 “concerned)

*Left-right self-placement*

“In politics sometimes people talk about "the left" and "the right". Where would you place yourself on a scale from 0 to 10 where 1 means "the left" and 10 means "the right"?”

Recoded: standardized scale

*Eurosceptic parties*

“Which party did you vote for in the last (NATIONALITY) general election in (YEAR)?”

| **Country** | **Eurosceptic parties** |
| --- | --- |
|  |  |
| France | Front National  Mouvement pour la France |
| Germany | Alternative fur Deutschland  Die Linke |
| Italy | Fratelli d’Italia  Lega Nord  Movimento 5 Stelle |
| Poland | KORWiN  Kukiz 15  Prawo i Sprawiedliwosc |
| Spain | Unidos-Podemos |
| Sweden | Sverigedemokraterna  Vensterpartiet |
|  |  |

## Descriptive statistics

#### Table A1. Descriptive statistics of predictors

|  |  |  |  |  |  |  |
| --- | --- | --- | --- | --- | --- | --- |
| **Variable** | **Obs.** | **Mean** | **St. Dev.** | **Min.** | **Max.** | **Perc.** |
|  |  |  |  |  |  |  |
| Cross-national solidarity  Not an EU task  Not be provided  Offered voluntarily  With conditionality  As soft loans  Unconditional | 7,953 |  |  |  |  | 6.6  12.3  10.2  37.5  19.7  13.7 |
| Egotropic concerns  Not concerned  Concerned | 9,317 |  |  |  |  | 66.2  33.8 |
| Sociotropic concerns  Not concerned  Concerned | 9,296 |  |  |  |  | 52.8  47.2 |
| (std.) Left-right self-placement | 9,326 | 0 | 1 | -2.092 | 2.084 |  |
| Eurosceptic vote  No  Yes | 6,488 |  |  |  |  | 73.0  27.0 |
| Trans-EU experiences  No  Yes | 9,320 |  |  |  |  | 28.1  71.9 |
| Gender  Male  Female | 9,326 |  |  |  |  | 39.8  60.2 |
| Age  18-34  35-54  55+ | 9,326 |  |  |  |  | 43.7  24.1  32.2 |
| Education  Lower secondary or less  Upper secondary  Tertiary or more | 9,308 |  |  |  |  | 25.3  45.0  29.7 |
| Occupation  Employers and self employed  Salaried middle class  Socio cultural specialist  Service and production workers  Unemployed  Welfare recipients  Other inactive | 9,275 |  |  |  |  | 9.0  22.0  8.9  22.4  5.1  17.1  15.5 |
| Income | 7,845 | 3.43 | 2.37 | 1 | 12 |  |
|  |  |  |  |  |  |  |

*Source*: REScEU 2016 survey

#### Table A2. Frequency distribution of cross-national solidarity by country

|  |  |  |  |  |  |  |  |
| --- | --- | --- | --- | --- | --- | --- | --- |
|  | **Cross-national financial help in times of crisis** | | | | | |  |
|  | **Not an EU task** | **Not be provided** | **Offered voluntarily** | **With conditionality** | **As soft loans** | **Unconditional** | **Obs.** |
| **France** | 93 | 218 | 99 | 512 | 198 | 187 | 1,307 |
|  | 7.12 | 16.68 | 7.57 | 39.17 | 15.15 | 14.31 | 100.00 |
| **Germany** | 97 | 199 | 112 | 566 | 188 | 156 | 1,318 |
|  | 7.36 | 15.10 | 8.50 | 42.94 | 14.26 | 11.84 | 100.00 |
| **Italy** | 32 | 96 | 108 | 501 | 353 | 224 | 1,314 |
|  | 2.44 | 7.31 | 8.22 | 38.13 | 26.86 | 17.05 | 100.00 |
| **Poland** | 103 | 170 | 254 | 414 | 248 | 142 | 1,331 |
|  | 7.74 | 12.77 | 19.08 | 31.10 | 18.63 | 10.67 | 100.00 |
| **Spain** | 81 | 120 | 92 | 542 | 310 | 206 | 1,351 |
|  | 6.00 | 8.88 | 6.81 | 40.12 | 22.95 | 15.25 | 100.00 |
| **Sweden** | 117 | 176 | 150 | 448 | 267 | 174 | 1,332 |
|  | 8.78 | 13.21 | 11.26 | 33.63 | 20.05 | 13.06 | 100.00 |
| **Total** | 523 | 979 | 815 | 2983 | 1564 | 1089 | 7,953 |
|  | 6.58 | 12.31 | 10.25 | 37.51 | 19.67 | 13.69 | 100.00 |
|  |  |  |  |  |  |  |  |

*Note*: For each country, the first row reports frequencies and the second row reports row percentages.

*Source*: REScEU 2016 survey

## Regression models

#### Table A3. Regressions results with no interactions in the pooled sample

|  | (1) | (2) | (3) | (5) | (6) |
| --- | --- | --- | --- | --- | --- |
| VARIABLES | Not an EU task | Not be provided | Offered voluntarily | As soft loans | No conditions |
|  |  |  |  |  |  |
| Gender | -0.260** | -0.086 | -0.053 | -0.106 | -0.218*** |
|  | (0.111) | (0.086) | (0.095) | (0.072) | (0.081) |
| *Age* |  |  |  |  |  |
| 18-34=reference cat. |  |  |  |  |  |
| 35-54 | -0.362*** | 0.121 | -0.407*** | 0.296*** | -0.197* |
|  | (0.132) | (0.104) | (0.116) | (0.091) | (0.101) |
| 55+ | -0.986*** | -0.116 | -0.673*** | 0.485*** | -0.033 |
|  | (0.144) | (0.115) | (0.124) | (0.092) | (0.103) |
| *Education* |  |  |  |  |  |
| Lower secondary=reference cat. |  |  |  |  |  |
| Upper secondary | -0.600*** | -0.263** | -0.249** | -0.092 | -0.203** |
|  | (0.134) | (0.107) | (0.117) | (0.091) | (0.102) |
| Tertiary | -0.631*** | -0.558*** | -0.354*** | -0.229** | -0.426*** |
|  | (0.162) | (0.129) | (0.136) | (0.104) | (0.122) |
| *Occupation* |  |  |  |  |  |
| Salaried middle class=reference cat. |  |  |  |  |  |
| Employers and self emp. | 0.048 | 0.103 | 0.095 | 0.145 | 0.126 |
|  | (0.222) | (0.171) | (0.183) | (0.136) | (0.159) |
| Socio-cultural prof. | 0.337* | 0.265 | 0.249 | 0.234* | 0.319** |
|  | (0.203) | (0.164) | (0.178) | (0.136) | (0.157) |
| Service and production workers | -0.244 | 0.121 | -0.124 | -0.170 | 0.065 |
|  | (0.164) | (0.123) | (0.136) | (0.108) | (0.121) |
| Unemployed | -0.135 | -0.189 | -0.678** | -0.048 | 0.187 |
|  | (0.271) | (0.226) | (0.269) | (0.173) | (0.185) |
| Welfare recipients | 0.118 | 0.076 | 0.018 | -0.099 | -0.335** |
|  | (0.188) | (0.146) | (0.159) | (0.122) | (0.146) |
| Other inactive | -0.059 | -0.284* | -0.208 | 0.019 | 0.047 |
|  | (0.182) | (0.157) | (0.157) | (0.125) | (0.139) |
| Income | -0.019 | -0.026 | -0.052*** | -0.048*** | -0.065*** |
|  | (0.025) | (0.019) | (0.020) | (0.017) | (0.020) |
| *Country* |  |  |  |  |  |
| Germany=reference cat. |  |  |  |  |  |
| France | 0.329* | 0.380*** | 0.070 | 0.131 | 0.300** |
|  | (0.182) | (0.133) | (0.169) | (0.132) | (0.140) |
| Italy | -0.652*** | -0.515*** | 0.276* | 0.691*** | 0.476*** |
|  | (0.226) | (0.160) | (0.166) | (0.122) | (0.136) |
| Poland | 0.568*** | 0.291** | 1.236*** | 0.650*** | 0.309** |
|  | (0.183) | (0.144) | (0.148) | (0.132) | (0.152) |
| Spain | 0.036 | -0.335** | -0.199 | 0.547*** | 0.311** |
|  | (0.185) | (0.148) | (0.177) | (0.122) | (0.138) |
| Sweden | 0.696*** | 0.215 | 0.650*** | 0.553*** | 0.494*** |
|  | (0.175) | (0.140) | (0.156) | (0.126) | (0.140) |
| Constant | -0.655** | -0.695*** | -0.867*** | -0.847*** | -0.481** |
|  | (0.276) | (0.220) | (0.247) | (0.191) | (0.211) |
|  |  |  |  |  |  |
| Observations | 6,611 | 6,611 | 6,611 | 6,611 | 6,611 |

*Note*: category 4 “With conditions” of the dependent variable is the baseline category in the multinomial model. ***p<0.01 **p<0.05 *p<0.1

*Source*: REScEU 2016 survey

#### Table A4. Regression results of the interaction between egotropic economic concerns and country dummies

|  | (1) | (2) | (3) | (5) | (6) |
| --- | --- | --- | --- | --- | --- |
| VARIABLES | Not a EU task | Not be provided | Offered voluntarily | As soft loans | No conditions |
|  |  |  |  |  |  |
| Gender | -0.287** | -0.098 | -0.051 | -0.093 | -0.249*** |
|  | (0.114) | (0.087) | (0.096) | (0.073) | (0.082) |
| *Age* |  |  |  |  |  |
| 18-34=reference cat. |  |  |  |  |  |
| 35-54 | -0.417*** | 0.053 | -0.396*** | 0.302*** | -0.195* |
|  | (0.133) | (0.105) | (0.116) | (0.092) | (0.103) |
| 55+ | -0.947*** | -0.170 | -0.680*** | 0.489*** | -0.049 |
|  | (0.147) | (0.118) | (0.127) | (0.094) | (0.105) |
| *Education* |  |  |  |  |  |
| Lower secondary=ref. cat. |  |  |  |  |  |
| Upper secondary | -0.432*** | -0.196* | -0.144 | -0.069 | -0.137 |
|  | (0.140) | (0.110) | (0.121) | (0.093) | (0.105) |
| Tertiary | -0.356** | -0.458*** | -0.197 | -0.190* | -0.353*** |
|  | (0.173) | (0.134) | (0.142) | (0.107) | (0.127) |
| *Occupation* |  |  |  |  |  |
| Salaried middle class=ref. cat. |  |  |  |  |  |
| Employers and self emp. | 0.070 | 0.035 | 0.138 | 0.177 | 0.191 |
|  | (0.225) | (0.176) | (0.185) | (0.138) | (0.162) |
| Socio-cultural prof. | 0.345* | 0.266 | 0.241 | 0.220 | 0.368** |
|  | (0.206) | (0.166) | (0.179) | (0.138) | (0.158) |
| Service and production workers | -0.297* | 0.082 | -0.109 | -0.150 | 0.118 |
|  | (0.166) | (0.124) | (0.137) | (0.109) | (0.122) |
| Unemployed | -0.247 | -0.323 | -0.681** | -0.040 | 0.207 |
|  | (0.271) | (0.227) | (0.272) | (0.176) | (0.191) |
| Welfare recipients | 0.012 | 0.002 | -0.010 | -0.070 | -0.276* |
|  | (0.193) | (0.150) | (0.165) | (0.123) | (0.147) |
| Other inactive | -0.077 | -0.297* | -0.218 | 0.030 | 0.076 |
|  | (0.184) | (0.158) | (0.159) | (0.126) | (0.140) |
| Income | -0.002 | -0.011 | -0.050** | -0.045*** | -0.060*** |
|  | (0.025) | (0.019) | (0.021) | (0.017) | (0.020) |
| Trans-EU experiences | -0.872*** | -0.248** | -0.400*** | -0.075 | -0.227** |
|  | (0.120) | (0.097) | (0.103) | (0.084) | (0.094) |
| (Std.) Left-right self-placement | 0.179*** | 0.227*** | 0.076* | -0.159*** | -0.154*** |
|  | (0.053) | (0.043) | (0.046) | (0.034) | (0.041) |
| Egotropic economic concern | 0.518** | 0.625*** | -0.238 | 0.159 | -0.659** |
|  | (0.263) | (0.198) | (0.276) | (0.206) | (0.272) |
| *Country* |  |  |  |  |  |
| Germany=ref. cat. |  |  |  |  |  |
| France | 0.715*** | 0.361* | 0.192 | 0.307* | 0.606*** |
|  | (0.236) | (0.188) | (0.213) | (0.173) | (0.171) |
| Italy | -0.309 | -0.487** | 0.229 | 0.604*** | 0.039 |
|  | (0.288) | (0.219) | (0.201) | (0.152) | (0.175) |
| Poland | 0.841*** | 0.426** | 1.137*** | 0.745*** | 0.291* |
|  | (0.213) | (0.173) | (0.169) | (0.151) | (0.170) |
| Spain | 0.492** | -0.154 | -0.004 | 0.611*** | 0.317* |
|  | (0.234) | (0.201) | (0.212) | (0.151) | (0.166) |
| Sweden | 0.745*** | 0.297* | 0.733*** | 0.736*** | 0.517*** |
|  | (0.220) | (0.175) | (0.178) | (0.146) | (0.159) |
| Egotropic economic concern#France | -0.768** | -0.259 | -0.119 | -0.304 | -0.026 |
|  | (0.367) | (0.267) | (0.366) | (0.274) | (0.333) |
| Egotropic economic concern#Italy | -0.586 | -0.070 | 0.329 | 0.158 | 1.446*** |
|  | (0.477) | (0.323) | (0.363) | (0.260) | (0.329) |
| Egotropic economic concern#Poland | -0.677 | -0.355 | 0.525 | -0.083 | 0.748* |
|  | (0.446) | (0.332) | (0.359) | (0.308) | (0.387) |
| Egotropic economic concern#Spain | -0.623* | -0.275 | -0.210 | -0.223 | 0.487 |
|  | (0.374) | (0.295) | (0.390) | (0.258) | (0.327) |
| Egotropic economic concern#Sweden | 0.257 | -0.160 | -0.151 | -0.720** | 0.344 |
|  | (0.362) | (0.292) | (0.375) | (0.295) | (0.357) |
| Constant | -0.517* | -0.810*** | -0.680*** | -0.938*** | -0.339 |
|  | (0.298) | (0.242) | (0.257) | (0.206) | (0.223) |
|  |  |  |  |  |  |
| Observations | 6,526 | 6,526 | 6,526 | 6,526 | 6,526 |
|  |  |  |  |  |  |

*Note*: category 4 “With conditions” of the dependent variable is the baseline category in the multinomial model. ***p<0.01 **p<0.05 *p<0.1

*Source*: REScEU 2016 survey

#### Table A5. Regression results of the interaction between sociotropic economic concerns and country dummies

|  | (1) | (2) | (3) | (5) | (6) |
| --- | --- | --- | --- | --- | --- |
| VARIABLES | Not a EU task | Not be provided | Offered voluntarily | As soft loans | No conditions |
|  |  |  |  |  |  |
| Gender | -0.275** | -0.141 | -0.045 | -0.094 | -0.213*** |
|  | (0.114) | (0.088) | (0.096) | (0.073) | (0.082) |
| *Age* |  |  |  |  |  |
| 18-34=reference cat. |  |  |  |  |  |
| 35-54 | -0.381*** | 0.072 | -0.396*** | 0.308*** | -0.170* |
|  | (0.131) | (0.105) | (0.116) | (0.092) | (0.102) |
| 55+ | -0.947*** | -0.150 | -0.655*** | 0.503*** | -0.027 |
|  | (0.146) | (0.118) | (0.128) | (0.094) | (0.106) |
| *Education* |  |  |  |  |  |
| Lower secondary=ref. cat. |  |  |  |  |  |
| Upper secondary | -0.464*** | -0.212* | -0.139 | -0.068 | -0.131 |
|  | (0.138) | (0.110) | (0.120) | (0.093) | (0.105) |
| Tertiary | -0.356** | -0.468*** | -0.203 | -0.194* | -0.348*** |
|  | (0.170) | (0.134) | (0.142) | (0.107) | (0.127) |
| *Occupation* |  |  |  |  |  |
| Salaried middle class=ref. cat. |  |  |  |  |  |
| Employers and self emp. | 0.094 | 0.067 | 0.135 | 0.188 | 0.202 |
|  | (0.225) | (0.176) | (0.184) | (0.138) | (0.161) |
| Socio-cultural prof. | 0.337 | 0.308* | 0.237 | 0.212 | 0.328** |
|  | (0.206) | (0.167) | (0.180) | (0.138) | (0.158) |
| Service and production workers | -0.291* | 0.090 | -0.124 | -0.154 | 0.107 |
|  | (0.166) | (0.124) | (0.136) | (0.109) | (0.122) |
| Unemployed | -0.206 | -0.279 | -0.688** | -0.036 | 0.243 |
|  | (0.271) | (0.226) | (0.270) | (0.175) | (0.187) |
| Welfare recipients | 0.067 | 0.048 | -0.030 | -0.072 | -0.301** |
|  | (0.193) | (0.149) | (0.164) | (0.123) | (0.148) |
| Other inactive | -0.056 | -0.283* | -0.236 | 0.026 | 0.066 |
|  | (0.184) | (0.159) | (0.159) | (0.126) | (0.141) |
| Income | -0.010 | -0.020 | -0.049** | -0.045*** | -0.060*** |
|  | (0.025) | (0.019) | (0.021) | (0.017) | (0.020) |
| Trans-EU experiences | -0.878*** | -0.260*** | -0.400*** | -0.084 | -0.235** |
|  | (0.120) | (0.097) | (0.103) | (0.084) | (0.094) |
| (Std.) Left-right self-placement | 0.167*** | 0.222*** | 0.081* | -0.150*** | -0.152*** |
|  | (0.053) | (0.043) | (0.046) | (0.034) | (0.041) |
| Sociotropic economic concern | 0.094 | 0.881*** | -0.017 | 0.302 | -0.209 |
|  | (0.271) | (0.193) | (0.251) | (0.196) | (0.230) |
| *Country* |  |  |  |  |  |
| Germany=ref. cat. |  |  |  |  |  |
| France | 0.600** | 0.229 | 0.410 | 0.610*** | 0.981*** |
|  | (0.285) | (0.264) | (0.259) | (0.210) | (0.204) |
| Italy | -0.037 | -0.100 | 0.518** | 0.717*** | 0.393* |
|  | (0.322) | (0.266) | (0.238) | (0.188) | (0.210) |
| Poland | 0.649*** | 0.433** | 1.217*** | 0.823*** | 0.399** |
|  | (0.218) | (0.190) | (0.176) | (0.157) | (0.176) |
| Spain | 0.467** | -0.033 | -0.001 | 0.556*** | 0.464*** |
|  | (0.236) | (0.220) | (0.232) | (0.167) | (0.178) |
| Sweden | 0.637*** | 0.243 | 0.727*** | 0.763*** | 0.616*** |
|  | (0.224) | (0.197) | (0.190) | (0.154) | (0.168) |
| Sociotropic economic concern#France | -0.291 | -0.301 | -0.456 | -0.750*** | -0.700** |
|  | (0.390) | (0.311) | (0.363) | (0.284) | (0.307) |
| Sociotropic economic concern#Italy | -0.842* | -0.895*** | -0.299 | -0.210 | 0.327 |
|  | (0.474) | (0.336) | (0.349) | (0.264) | (0.307) |
| Sociotropic economic concern#Poland | 0.019 | -0.281 | 0.139 | -0.332 | 0.113 |
|  | (0.402) | (0.299) | (0.328) | (0.284) | (0.345) |
| Sociotropic economic concern#Spain | -0.466 | -0.587** | -0.225 | -0.178 | -0.083 |
|  | (0.378) | (0.296) | (0.366) | (0.252) | (0.294) |
| Sociotropic economic concern#Sweden | 0.456 | -0.171 | -0.076 | -0.607** | -0.085 |
|  | (0.362) | (0.280) | (0.333) | (0.270) | (0.312) |
| Constant | -0.368 | -0.850*** | -0.738*** | -0.981*** | -0.466** |
|  | (0.290) | (0.236) | (0.258) | (0.208) | (0.226) |
|  |  |  |  |  |  |
| Observations | 6,521 | 6,521 | 6,521 | 6,521 | 6,521 |
|  |  |  |  |  |  |

*Note*: category 4 “With conditions” of the dependent variable is the baseline category in the multinomial model. ***p<0.01 **p<0.05 *p<0.1

*Source*: REScEU 2016 survey

#### Table A6. Regression results of the interaction between left-right self-placement and country dummies

|  | (1) | (2) | (3) | (5) | (6) |
| --- | --- | --- | --- | --- | --- |
| VARIABLES | Not a EU task | Not be provided | Offered voluntarily | As soft loans | No conditions |
|  |  |  |  |  |  |
| Gender | -0.286** | -0.137 | -0.114 | -0.032 | -0.185* |
|  | (0.138) | (0.102) | (0.113) | (0.085) | (0.096) |
| *Age* |  |  |  |  |  |
| 18-34=reference cat. |  |  |  |  |  |
| 35-54 | -0.387** | -0.001 | -0.438*** | 0.350*** | -0.091 |
|  | (0.162) | (0.124) | (0.138) | (0.109) | (0.120) |
| 55+ | -0.982*** | -0.174 | -0.703*** | 0.486*** | -0.123 |
|  | (0.173) | (0.137) | (0.149) | (0.109) | (0.123) |
| *Education* |  |  |  |  |  |
| Lower secondary=ref. cat. |  |  |  |  |  |
| Upper secondary | -0.497*** | -0.317** | -0.243* | -0.069 | -0.194 |
|  | (0.170) | (0.133) | (0.146) | (0.111) | (0.126) |
| Tertiary | -0.393* | -0.572*** | -0.208 | -0.160 | -0.431*** |
|  | (0.205) | (0.160) | (0.166) | (0.125) | (0.148) |
| *Occupation* |  |  |  |  |  |
| Salaried middle class=ref. cat. |  |  |  |  |  |
| Employers and self emp. | -0.222 | 0.098 | -0.015 | 0.063 | 0.062 |
|  | (0.279) | (0.194) | (0.217) | (0.155) | (0.183) |
| Socio-cultural prof. | 0.251 | 0.138 | 0.239 | 0.099 | 0.194 |
|  | (0.237) | (0.189) | (0.204) | (0.154) | (0.181) |
| Service and production workers | -0.499** | 0.024 | -0.175 | -0.370*** | -0.094 |
|  | (0.204) | (0.146) | (0.162) | (0.127) | (0.142) |
| Unemployed | -0.363 | -0.238 | -1.272*** | -0.295 | -0.287 |
|  | (0.360) | (0.293) | (0.417) | (0.227) | (0.267) |
| Welfare recipients | -0.089 | -0.037 | -0.120 | -0.186 | -0.415** |
|  | (0.225) | (0.173) | (0.190) | (0.137) | (0.169) |
| Other inactive | -0.013 | -0.427** | -0.332* | -0.204 | 0.033 |
|  | (0.227) | (0.202) | (0.198) | (0.154) | (0.168) |
| Income | -0.018 | 0.001 | -0.047** | -0.054*** | -0.061*** |
|  | (0.029) | (0.022) | (0.024) | (0.020) | (0.023) |
| Trans-EU experiences | -0.909*** | -0.198* | -0.397*** | -0.083 | -0.193* |
|  | (0.149) | (0.117) | (0.125) | (0.100) | (0.115) |
| (Std.) Left-right self-placement | -0.370 | -0.117 | -0.062 | -0.364*** | -0.593*** |
|  | (0.237) | (0.135) | (0.148) | (0.135) | (0.178) |
| Eurosceptic vote | 0.561*** | 0.683*** | 0.554*** | 0.300*** | 0.566*** |
|  | (0.151) | (0.114) | (0.122) | (0.096) | (0.106) |
| *Country* |  |  |  |  |  |
| Germany=ref. cat. |  |  |  |  |  |
| France | 0.704*** | 0.521*** | 0.048 | 0.123 | 0.699*** |
|  | (0.247) | (0.180) | (0.222) | (0.164) | (0.185) |
| Italy | -0.379 | -0.297 | 0.338 | 0.625*** | 0.538*** |
|  | (0.291) | (0.196) | (0.205) | (0.151) | (0.185) |
| Poland | 0.683*** | 0.161 | 1.116*** | 0.592*** | 0.376* |
|  | (0.251) | (0.188) | (0.183) | (0.156) | (0.198) |
| Spain | 0.404 | -0.182 | -0.082 | 0.520*** | 0.496*** |
|  | (0.249) | (0.188) | (0.216) | (0.147) | (0.184) |
| Sweden | 1.069*** | 0.289* | 0.620*** | 0.553*** | 0.709*** |
|  | (0.232) | (0.172) | (0.187) | (0.146) | (0.179) |
| (Std.) Left-right#France | 0.482* | 0.490*** | 0.082 | 0.043 | 0.260 |
|  | (0.275) | (0.166) | (0.204) | (0.164) | (0.200) |
| (Std.) Left-right#Italy | 0.000 | 0.000 | 0.000 | 0.000 | 0.000 |
|  | (0.000) | (0.000) | (0.000) | (0.000) | (0.000) |
| (Std.) Left-right#Poland | 0.387 | 0.282 | 0.229 | 0.348** | 0.482** |
|  | (0.309) | (0.188) | (0.198) | (0.159) | (0.208) |
| (Std.) Left-right#Spain | 0.655** | 0.223 | 0.209 | 0.134 | 0.560** |
|  | (0.278) | (0.187) | (0.193) | (0.168) | (0.225) |
| (Std.) Left-right#Sweden | 0.386 | 0.097 | 0.062 | 0.119 | 0.455** |
|  | (0.272) | (0.193) | (0.205) | (0.159) | (0.205) |
| Constant | -0.537 | -0.776*** | -0.691** | -0.889*** | -0.680** |
|  | (0.340) | (0.268) | (0.300) | (0.233) | (0.267) |
|  |  |  |  |  |  |
| Observations | 4,741 | 4,741 | 4,741 | 4,741 | 4,741 |
|  |  |  |  |  |  |

*Note*: category 4 “With conditions” of the dependent variable is the baseline category in the multinomial model. ***p<0.01 **p<0.05 *p<0.1

*Source*: REScEU 2016 survey

#### Table A7. Regression results of the interaction between Eurosceptic vote choices and country dummies

|  | (1) | (2) | (3) | (5) | (6) |
| --- | --- | --- | --- | --- | --- |
| VARIABLES | Not a EU task | Not be provided | Offered voluntarily | As soft loans | No conditions |
|  |  |  |  |  |  |
| Gender | -0.288** | -0.118 | -0.112 | -0.019 | -0.191** |
|  | (0.137) | (0.102) | (0.113) | (0.084) | (0.096) |
| *Age* |  |  |  |  |  |
| 18-34=reference cat. |  |  |  |  |  |
| 35-54 | -0.395** | -0.030 | -0.431*** | 0.346*** | -0.080 |
|  | (0.161) | (0.124) | (0.139) | (0.109) | (0.120) |
| 55+ | -0.999*** | -0.217 | -0.707*** | 0.460*** | -0.113 |
|  | (0.173) | (0.138) | (0.150) | (0.109) | (0.123) |
| *Education* |  |  |  |  |  |
| Lower secondary=ref. cat. |  |  |  |  |  |
| Upper secondary | -0.492*** | -0.305** | -0.244* | -0.072 | -0.198 |
|  | (0.170) | (0.133) | (0.146) | (0.111) | (0.126) |
| Tertiary | -0.402* | -0.576*** | -0.207 | -0.181 | -0.442*** |
|  | (0.205) | (0.159) | (0.166) | (0.125) | (0.149) |
| *Occupation* |  |  |  |  |  |
| Salaried middle class=ref. cat. |  |  |  |  |  |
| Employers and self emp. | -0.189 | 0.120 | 0.006 | 0.102 | 0.096 |
|  | (0.280) | (0.195) | (0.217) | (0.155) | (0.184) |
| Socio-cultural prof. | 0.260 | 0.142 | 0.246 | 0.108 | 0.207 |
|  | (0.238) | (0.190) | (0.204) | (0.154) | (0.181) |
| Service and production workers | -0.487** | 0.025 | -0.168 | -0.350*** | -0.069 |
|  | (0.205) | (0.145) | (0.162) | (0.127) | (0.142) |
| Unemployed | -0.352 | -0.188 | -1.236*** | -0.258 | -0.300 |
|  | (0.363) | (0.292) | (0.418) | (0.228) | (0.266) |
| Welfare recipients | -0.078 | -0.021 | -0.110 | -0.177 | -0.406** |
|  | (0.226) | (0.173) | (0.190) | (0.137) | (0.168) |
| Other inactive | 0.015 | -0.412** | -0.321 | -0.176 | 0.070 |
|  | (0.228) | (0.200) | (0.199) | (0.154) | (0.168) |
| Income | -0.019 | 0.001 | -0.048** | -0.053*** | -0.060*** |
|  | (0.029) | (0.022) | (0.024) | (0.020) | (0.023) |
| Trans-EU experiences | -0.879*** | -0.162 | -0.384*** | -0.071 | -0.187 |
|  | (0.149) | (0.117) | (0.125) | (0.100) | (0.115) |
| (Std.) Left-right self-placement | 0.124* | 0.174*** | 0.061 | -0.174*** | -0.163*** |
|  | (0.065) | (0.049) | (0.055) | (0.040) | (0.047) |
| Eurosceptic vote | 0.172 | 0.863*** | 0.746** | 0.377 | 0.439 |
|  | (0.441) | (0.276) | (0.322) | (0.282) | (0.309) |
| *Country* |  |  |  |  |  |
| Germany=ref. cat. |  |  |  |  |  |
| France | 0.539* | 0.544*** | 0.018 | 0.174 | 0.748*** |
|  | (0.278) | (0.202) | (0.265) | (0.176) | (0.191) |
| Italy | -0.521 | -0.099 | 0.426* | 0.680*** | 0.346 |
|  | (0.399) | (0.248) | (0.257) | (0.171) | (0.213) |
| Poland | 0.732** | 0.416* | 1.118*** | 0.690*** | 0.268 |
|  | (0.298) | (0.233) | (0.232) | (0.183) | (0.238) |
| Spain | 0.211 | -0.035 | -0.033 | 0.478*** | 0.271 |
|  | (0.273) | (0.209) | (0.249) | (0.159) | (0.194) |
| Sweden | 0.834*** | 0.099 | 0.684*** | 0.454*** | 0.556*** |
|  | (0.251) | (0.206) | (0.215) | (0.161) | (0.189) |
| Eurosceptic vote #France | 0.453 | 0.133 | -0.060 | -0.197 | -0.490 |
|  | (0.558) | (0.362) | (0.476) | (0.395) | (0.425) |
| Eurosceptic vote #Italy | 0.333 | -0.551 | -0.336 | -0.319 | 0.212 |
|  | (0.654) | (0.397) | (0.427) | (0.343) | (0.382) |
| Eurosceptic vote #Poland | 0.145 | -0.661* | -0.130 | -0.311 | 0.118 |
|  | (0.541) | (0.378) | (0.395) | (0.354) | (0.406) |
| Eurosceptic vote #Spain | 0.642 | -0.449 | -0.189 | 0.116 | 0.356 |
|  | (0.565) | (0.412) | (0.466) | (0.346) | (0.384) |
| Eurosceptic vote #Sweden | 0.849 | 0.494 | -0.214 | 0.328 | 0.330 |
|  | (0.521) | (0.367) | (0.424) | (0.356) | (0.392) |
| Constant | -0.414 | -0.846*** | -0.739** | -0.912*** | -0.573** |
|  | (0.350) | (0.276) | (0.308) | (0.239) | (0.263) |
|  |  |  |  |  |  |
| Observations | 4,741 | 4,741 | 4,741 | 4,741 | 4,741 |
|  |  |  |  |  |  |

*Note*: category 4 “With conditions” of the dependent variable is the baseline category in the multinomial model. ***p<0.01 **p<0.05 *p<0.1

*Source*: REScEU 2016 survey

## Robustness checks (results from models in single countries)

#### Figure A2. Association between egotropic economic concerns and EU financial support

*Source*: Authors’ elaboration on REScEU 2016 survey data.

#### Figure A3. Association between sociotropic economic concerns and EU financial support

*Source*: Authors’ elaboration on REScEU 2016 survey data.

#### Figure A4. Association between ideological predispositions and EU financial support

*Source*: Authors’ elaboration on REScEU 2016 survey data.

#### Figure A5. Association between Eurosceptic vote choice and EU financial support

*Source*: Authors’ elaboration on REScEU 2016 survey data.

## References

Erikson, R. and Goldthorpe, J. H. (1992) *The Constant Flux: A Study of Class Mobility in Industrial Societies*, New York: Oxford University Press.

Kitschelt, H. and Rehm, P. (2014) ‘Occupations as a Site of Political Preference Formation’, *Comparative Political Studies* 47(12): 1670–1706.

Lepsius, M. R. (2009) *Interessen, Ideen und Institutionen*, 2nd Ed., Wiesbaden: Springer VS.

Oesch, D. (2006) *Redrawing the class map: Stratification and institutions in Britain, Germany, Sweden and Switzerland*, Basingstoke: Palgrave Macmillan.

Oesch, D. (2008) ‘Explaining Workers’ Support for Right-Wing Populist Parties in Western Europe: Evidence from Austria, Belgium, France, Norway, and Switzerland’, *International Political Science Review* 29(3): 349–373.
